# Hearing loss and its association with occupational noise exposure among Saudi dentists: a cross-sectional study

**DOI:** 10.1038/bdjopen.2016.6

**Published:** 2016-11-04

**Authors:** Bander M Alabdulwahhab, Raneem I Alduraiby, May A Ahmed, Lamya I Albatli, Maram S Alhumain, Nada A Softah, Shaza Saleh

**Affiliations:** 1 Restorative Department, Royal Clinics Department of Dental Services, Part Time Faculty at Riyadh Colleges of Dentistry and Pharmacy, Riyadh, Saudi Arabia; 2 Riyadh Colleges of Dentistry and Pharmacy, Riyadh, Saudi Arabia; 3 King Faisal Specialist Hospital and Research Centre, Riyadh, Saudi Arabia

## Abstract

**Objectives/Aims:**

Dental practitioners are prone to hearing loss due to noise exposure encountered in dental clinics. The aim of this study was to determine whether the persistent high-frequency sounds produced by the dental equipment could cause hearing decrement among the Saudi dental practitioners.

**Materials and Methods:**

This cross-sectional study included 38 randomly selected Saudi dentists from different specialties who were exposed to noise during working hours and 38 individuals as a control group. The participants underwent four audiometric tests that included an otoscopic examination, tympanometry, pure tone audiometry and the distortion product otoacoustic emissions (DPOAE) test.

**Results:**

The data revealed that ~15.8% of the dentists and 2.6% of the control group had some hearing loss. No significant difference was found between the two groups in the pure tone audiometry test; however, qualitative analysis revealed a higher percentage of hearing loss among the dentists’ group as compared with their control counterparts. A statistically significant difference was found in DPOAEs between the two groups in the left ear (*P*=0.002), and between the right and left ears (*P*=0.005).

**Discussion:**

In the present cross-sectional study, the prevalence of hearing loss among dentists as assessed with the pure tone audiometry test was 15.8%. Which was in accordance with a previous study performed by Khaimook *et al*., which revealed the prevalence of hearing loss in dental personnel to be 17.7%; however, no significant differences were observed compared to the control group in both studies. The otoacoustic emission test in the left ear exhibited significant changes. These changes could have been due to the presence and continuity of the sounds produced by high- and low-velocity suction devices on the left side of the dental unit knowing that 97% of the dentists are right handed.

**Conclusion:**

Evidence suggests that noise from dental clinics can cause hearing problems, which had a greater effect on the left ear than the right; however, these problems are not severe in nature. Noise-induced hearing loss was more prevalent among the dentists than the control group.

## Introduction

According to the national institute for occupational safety and health, noise has been identified as one of the 10 leading causes of work-related diseases or injuries.^1^ The amount of damage depends primarily on the intensity of the noise and the duration of the exposure. Noise-induced hearing loss can be temporary following short-term exposure to noise, with the return of normal hearing after a period of rest.^2^ Injury to the ear due to noise occurs in two different manners that depend on the type of exposure. High-level short-duration exposures to more than 140 decibel (dB, i.e., a unit that measures sound intensity) can stretch the delicate inner ear tissues beyond their elastic limits and then rip or tear them apart. This type of damage (acoustic trauma) develops rapidly and causes an immediate and permanent hearing loss. The second type of injury occurs because of exposure to noise between 90 and 140 dB, which causes metabolic rather than mechanical damage to the cochlea, and this damage is related to the level and duration of exposure.^3^ The factors that affect the degree and extent of hearing impairment include the intensity and type of noise, the period of exposure each day, total work duration, distance from the source, and individual age and susceptibility.^4^

The Occupational Safety and Health Administration of the United State Department of Labor demands that employers develop and implement a noise-monitoring programme when employees are exposed to noise equal to or exceeding 85 dB for more than eight working hours. If this situation occurs, Occupational Safety and Health Administration requires employers to inform employees to establish and maintain an audiometric testing protocol, and to train workers how to prevent occupational hearing loss. When hazardous noise have not yet been eliminated, Occupational Safety and Health Administration also requires employers to provide hearing protection and to ensure that the workers utilise that protection.^2^

Pure tone audiometry is generally the first quantitative hearing test that is performed to assess the nature and degree of hearing loss in adults and children over 4 years of age to properly plan the most appropriate intervention because this test determines the faintest tones a person can hear at selected frequencies from low to high.^5,6^

Otoacoustic emissions (OAEs) permit the early detection of inner ear abnormalities that are associated with a wide variety of diseases and disorders, including non-pathologic etiologies, such as noise exposure and aging. Changes in outer hair cell length generate energy within the cochlea that contributes to hearing sensitivity and the ability to distinguish small differences in the frequencies of sounds.^7^

People are accustomed to everyday normal noise that is constantly present all around them. Similar to other working professionals, dental practitioners are exposed to many occupational hazards; hearing loss is definitely one such occupational hazard due to the noise that is constantly present during their work.^8^ Instruments in the dental office, such as high-speed turbine hand pieces, low-speed hand pieces and high-velocity suction devices, produce dangerously loud noises that may contribute to hearing loss. Long-term exposure to noise levels of greater than 80–85 dB is associated with an increased risk of hearing loss.^9^ Kilpatrick^10^ provided a list of the dB ratings of different office instruments and equipment and reported levels of 70–92 dB for high-speed turbine hand pieces, 86 dB for ultrasonic scalers and 74 dB for low-speed hand pieces.

A previous study by Alwazzan *et al.*^11^ that sought to determine the prevalence of hearing problems among dentists in Saudi Arabia concluded that all dental personnel exhibit roughly the same incidence of symptoms, which include tinnitus, speech discrimination difficulties and difficulties with speech discrimination in the presence of background noise; moreover, dental technicians were found to be the most affected group.

The aim of this study was to determine whether the persistent high-frequency sounds produced by the dental equipment could cause hearing decrement among the Saudi dental practitioners.

## Materials and methods

### Study group

This cross-sectional study was conducted from March to December 2015. Thirty-eight dentists from different specialties who work at various governmental hospitals and private clinics that were exposed to occupational noise and a control group of thirty-eight matched non-dental professionals were recruited; matching was done based on age, gender and whether that participant was a smoker/non-smoker. Both sample groups were selected randomly while observing the matching criteria for the control group only

The inclusion criteria for the experimental group included dentists who had been practicing dentistry for more than 5 years, which includes 2 years of preclinical practice. For the control group, the inclusion criterion was individuals who had not been exposed to noise during work hours. Both sample groups included individuals between the ages of 25 and 40 years. The exclusion criteria for both groups included daily loud music exposure for more than 3 h,^12^ history of chronic ear disease, ear surgery, ear trauma, ototoxic drugs, diabetes, previous sensorineural hearing loss, any hereditary factors and treatment with radiation or chemotherapy.

### Sample design

This study has received a formal review and approval from the ethics committee of Riyadh Colleges of Dentistry and Pharmacy. The potential participants were approached either by telephone or in person to explain the effects of instrument noise on hearing and were asked to be part of this cross-sectional study. After providing written informed consent, the participants underwent an otoscopic examination and tympanometry by an audiology specialist at Magrabi Hospitals and Centers. If abnormal findings were observed, the individual was excluded from further testing. Subsequently, the participants who fulfilled the inclusion criteria completed a demographic questionnaire and underwent pure tone audiometry and distortion product otoacoustic emission (DPOAE) testing.

The collected data included age group (25–28, 29–32, 33–36 and 37–40 years of age), gender, years of practice (5–8, 9–12 or ⩾13 years), actual number of hours exposed to loud noise each week (⩽27, 28–36 or ⩾37 h), days of exposure per week (3, 4, 5 or 6 days) and whether the dentists are left-handed or right-handed (Table 1).

### Test battery

#### Pure tone audiometry

**Figure 1:**
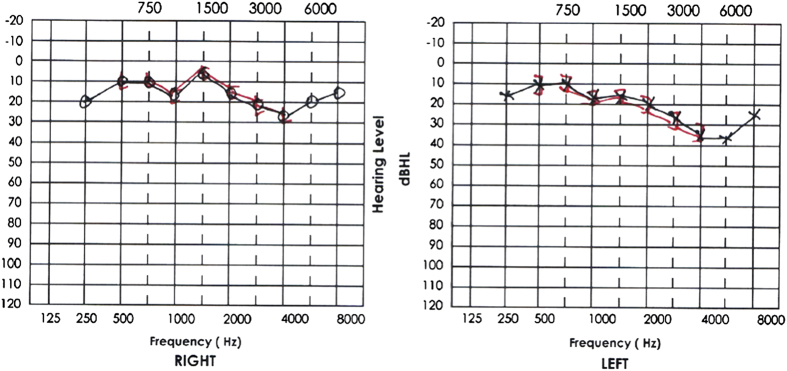
Audiograms showing results more than 25 dB in high frequencies in the right and left ear.

The audiometric examinations were performed using an audiometer (GSI 61, Grason Stadler, Minneapolis, MN, USA) in a 2×2 m double-walled sound booth that was calibrated according to the standards of the International Standard Organization (1964). Air conduction hearing thresholds were measured by pure tone audiometry at the following frequencies: 250 Hz, 500 Hz, 750 Hz, 1 kHz, 2 kHz, 3 kHz, 4 kHz, 6 kHz and 8 kHz. The pure tone averages at 4, 6 and 8 kHz reflect the frequency range that is most susceptible to noise-induced hearing loss. Losses of more than 25 dB in these frequencies are usually considered abnormal (see Figure 1).

#### Distortion Product Otoacoustic Emissions

Otoacoustic emission tests (DPOAE, OAE System, Pleasanton, CA, USA) are used to determine cochlear status and particularly hair cell function.^13^

### Statistical analysis

The IBM SPSS computer software (Statistical Package for the Social Sciences, version 20.0, SPSS Inc., Chicago, IL, USA) for Windows was used to perform the analyses. The 3Power G3.1 software (G*Power: Statistical Power Analyses Heinrich-Heine-University Düsseldorf, Düsseldorf, Germany) was used to conduct power analysis and determine the number of the required sample size, which was 25. Wilcoxon test was used to determine whether the differences in the pure tone audiometry and OAE test results were significantly different between the dentists and the control group. The level of significance was defined as equal to or less than 0.05.

**Figure 2:**
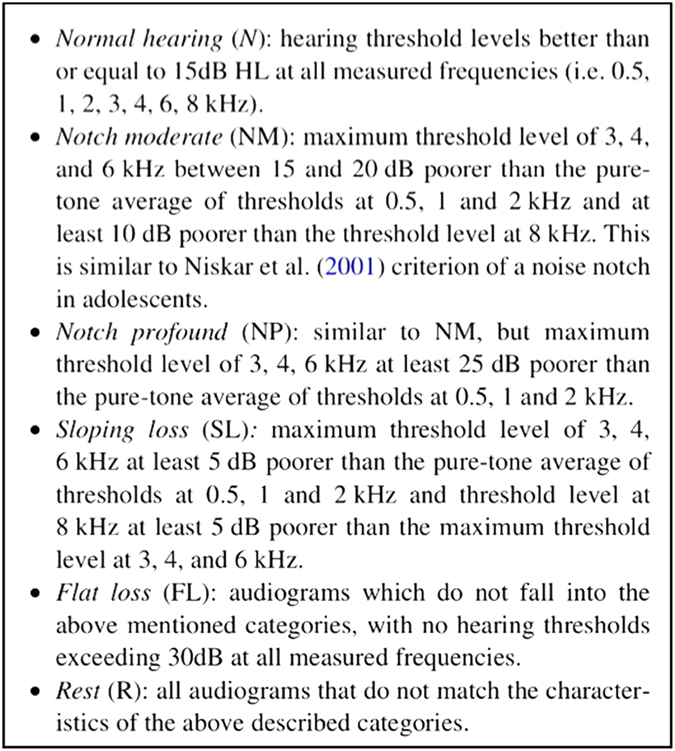
Criteria for the characterization of the pure-tone audiograms and the degree of the noise notch adapted from Jensen *et al.*^14^

Qualitative analysis of pure tone audiometry results was conducted based on the characterisation criteria proposed by Jensen *et al.*,^14^ which was used to investigate noise-induced hearing loss among musicians of symphony orchestras. They adopted a rather strict criterion for normal hearing, and more specific criteria for the degree of the noise notch if present (see Figure 2).

## Results

The ages of the 38 dentists and the 38 participants of the control group ranged from 25 to 40 years, and the sample included 23 (61%) males and 15 (39%) females (Table 1). The prevalence of hearing loss (as assessed by any decrease in hearing of more than 25 dB HL in the pure tone audiometry tests) was six participants in the group of 38 dentists (15.8%) and one participant in the control group of 38 participants (2.6%). When the more stringent criteria of 15 dB hearing loss (HL)^14^ was applied, it revealed that 29 of the dentists (76%) and 23 participants from the control group (60%) had some type of hearing loss.

No significant difference was observed between the two groups in the pure tone audiometry results at the following frequencies: 500 Hz, 1 kHz, 2 kHz, 4 kHz, 6 kHz, and 8 kHz. Moreover, there were no significant differences between dentists and the matched control group in terms of noise-induced hearing loss in the right and left ears separately or in both ears combined (Table 2).

The assessments of noise-induced hearing loss with the DPOAE tests revealed no significant differences between the two groups in the right ear (*P*=0.355), but a significant difference was found for the left ear (*P*=0.002) and in both ears combined (*P*=0.005) (Table 2). The mean values for the pure tone audiometry and the DPOAE tests for both dentists and their control counterparts are illustrated in Tables 3 and 4.

Qualitative analysis of the audiograms as proposed by Jensen *et al.*^14^ showed different patterns among the dentists as compared to their control counterparts. For the right ear, 5.2% of the dentists exhibited sloping loss, and 60.5% had flat loss. For the left ear, 7.8% had a moderate notch, and 50% had flat loss. On the other hand, 42.1% of the control group showed normal hearing in the right ear, and 52.6% had normal hearing in the left ear (see Table 5).

## Discussion

Occupational noise-induced hearing loss is defined as bilateral sensorineural hearing loss that develops gradually over a period of several years because of exposure to continuous or intermittent loud noise in the work place.^15^

Hearing loss due to aging or genetic factors is not preventable. In contrast, noise-induced hearing loss can be prevented by the use of protective equipment in noisy environments, including ear plugs and ear muffs.^16^ Unfortunately, none of the participants in this study were using any type of ear protection, potentially due to discomfort, fear that the protective device may interfere with communication, inconvenience, negative feedback from co-workers or patients and the belief that noise levels from dental instruments will not damage their hearing.^16^

A study that sought to determine the prevalence of hearing problems among dentists in Saudi Arabia concluded that all dental personnel exhibit roughly similar incidences of symptoms; i.e., 16.6% had tinnitus, 14.7% had speech discrimination difficulties and 63% had problems with speech discrimination in the presence of background noise. The incidences of symptom were similar because all dental personnel are exposed to similar noise levels.^11^

The occurrence of hearing loss due to prolonged exposure to noise levels greater than 85 dB without the use of any type of ear protection is well documented in the literature.^17–19^ Therefore, the noise generated in the dental clinic should not be underestimated.^20^ The sources of dental sounds that can be treated as potentially damaging to hearing include high-speed turbine hand pieces, high-velocity suction devices, ultrasonic scalers and other mixing devices.^8^

Altinoz *et al.*^21^ noted that personnel who work in noisy environments should not engage in noisy activities immediately following the workday. These authors stated that ‘the ear begins to recover its hearing ability when it is allowed to rest’.

The prevalence of noise-induced hearing loss among dental personnel has been reported to range from 7 to 16% in the literature.^22–24^ The study performed by Khaimook *et al.*^15^ revealed the prevalence of hearing loss in dental personnel to be 17.7%; however, no significant differences were observed compared with the control group.

In the present cross-sectional study, the prevalence of hearing loss among dentists as assessed with the pure tone audiometry test was 15.8%, which did not significantly differ from the results observed in the control group. Prevalence with more stringent criteria among dentists was 76%. A significant difference may have been observed with a larger sample. Regarding the DPOAEs that were used to compare the two groups, the left ear exhibited significant changes that could have been due to changes in the outer hair cell lengths. These changes could also have been due to the presence and continuity of the sounds produced by high- and low-velocity suction devices on the left side of the dental unit, considering that 97% of the dentists in this study were right-handed. Undoubtedly, the degree of risk to the dental practitioner depends upon certain factors such as the intensity of the sound and the duration of exposure.^8,25^

In an article written by Khaimook *et al.*,^15^ the authors stated that risk factors including the years of experience and the working hours per week influence hearing. Further study is recommended in the future to reveal risk factors related to dental specialty, working hours and years of experience.

To decrease the risk of developing noise-induced hearing loss, dental practitioners are encouraged to follow the recommendation of the ADA council on dental materials and devices, which include the following:

Preventive measures for noise attenuation should be directed in three areas: optimum maintenance of rotary equipment, reduction of the ambient noise level in the operatory and personal protection through the use of ear plugs.^26^

Dentists are advised to perform regular annual audiometry check-ups.^26^ This regular testing should identify those who have begun to lose their hearing before they acquire significant auditory impairments.^4,21,27^

It is necessary to produce dental hand pieces with additional noise control. Manufactures are urged to improve quality in terms of decreasing the sound levels produced by high-speed dental hand pieces. Furthermore, friction increases in old and worn machinery, which results in increases in sound levels and highlights the importance of maintenance and periodic replacement.^27^ During the construction and design of the dental clinic, consideration of the use of sound-absorbing materials is also recommended to decrease the noise level.^21^

Continuing education programmes would be beneficial in terms of decreasing the risk of noise-induced hearing loss among dental personnel. Moreover, dental school curricula ought to include education about the different occupational hazards.^11^

## Conclusion

Within the limitations of this study, evidence suggests that noise from dental clinics can cause hearing problems, which had a greater effect on the left ear than the right; however, these problems are not severe in nature. Noise-induced hearing loss was more prevalent among the dentists than the control group.

## Figures and Tables

**Table 1:** Participants’ demographic data

*Demographics*	n*=38; %
*Gender*	
Female	15 (39.47)
Male	23 (60.52)
	
*Age (years)*	
25–28	17 (44.73)
29–32	11 (28.94)
33–36	6 (15.78)
37–40	4 (10.52)
	
*Working days per week*	
3	2 (5.26)
4	11 (28.94)
5	19 (50)
6	6 (15.78)
	
*Working hours per week (h)*	
⩽18–27	13 (34.21)
28–36	11 (28.94)
⩾37 h	14 (36.84)
	
*Years of experience*	
5–8	19 (50)
9–12	11 (28.94)
⩾13	8 (21.05)
	
*Handiness*	
Right-handed	37 (97%)
Left-handed	1 (3%)

Abbreviation: n*, number of dentists.

**Table 2:** The difference between the dentists and the control group in the pure tone audiometry and (DPOAE) results

*Ear of participant*	*Dentist (*n*=38)	*Control (*n*=38)	*Pure tone audiometry*		*Otoacoustic emission*
			P*-value		P*-value
			*Low frequency*	*High frequency*	
Right	38	38	0.387	0.321	0.355
Left	38	38	0.293	0.217	0.003
Both ears (right and left)	38	38	0.173	0.132	0.005

Abbreviations: DOAE, distortion product otoacoustic emission; n*, number of participants; P*, level of significance<0.05.

**Table 3:** Illustrates descriptive statistics of all the variables for the dentists

*Ear of participant*	*Dentist (*n*=38)	*Pure tone audiometry*				*Otoacoustic emission*	
		*Low frequency*		*High frequency*		*Std*	*Mean*
		*Std*	*Mean*	*Std*	*Mean*		
Right	38	5.96077	11.2916	6.92840	11.6429	8.11467	12.6194
Left	38	6.24268	9.9379	8.00434	11.5600	7.95475	13.5362
Both ears (right and left)	38	6.10070	10.6147	7.43577	11.6014	8.04158	13.0778

Abbreviation: n*, number of dentists.

**Table 4:** Illustrates descriptive statistics of all the variables for the control group

*Ear of participant*	*Control (*n*=38)	*Pure tone audiometry*				*Otoacoustic emission*	
		*Low frequency*		*High frequency*		*Std*	*Mean*
		*Std*	*Mean*	*Std*	*Mean*		
Right	38	5.00613	10.4055	4.85405	10.0229	8.16295	13.2664
Left	38	5.45226	8.4011	7.11611	9.2416	7.68386	14.9819
Both ears (right and left)	38	5.29593	9.4033	6.06303	9.6322	7.96688	14.1242

Abbreviation: n*, number of control.

**Table 5:** The audiogram results for both groups according to the criteria proposed by Jensen *et al.*^14^ for characterisation of the pure tone audiograms

*Ear of participant*	*Dentist,* n*=38 (%)*						*Control,* n*=38 (%)*					
	*Normal*	*Notch moderate*	*Notch profound*	*Sloping loss*	*Flat loss*	*Rest*	*Normal*	*Notch moderate*	*Notch profound*	*Sloping loss*	*Flat loss*	*Rest*
Right ear	13 (34.2%)	0 (0%)	0 (0%)	2 (5.2%)	23 (60.5%)	0 (0%)	16 (42.1%)	0 (0%)	0 (0%)	1 (2.6%)	21 (55.2%)	0 (0%)
Left ear	15 (39.4%)	3 (7.8%)	0 (0%)	0 (0%)	19 (50%)	0 (0%)	20 (52.6%)	0 (0%)	0 (0%)	0 (0%)	18 (47.3%)	0 (0%)
